# Retrospective review of the activity and safety of apatinib and anlotinib in patients with advanced osteosarcoma and soft tissue sarcoma

**DOI:** 10.1007/s10637-020-00912-7

**Published:** 2020-02-25

**Authors:** Zhichao Tian, Huimin Liu, Fan Zhang, Liangyu Li, Xinhui Du, Chao Li, Jinpo Yang, Jiaqiang Wang

**Affiliations:** 1 Department of Orthopedics, The Affiliated Cancer Hospital of Zhengzhou University and Henan Cancer Hospital, Zhengzhou, 450008 Henan Province China; 2 Department of Medical Oncology, The Affiliated People’s Hospital of Zhengzhou University, Zhengzhou, 450003 Henan Province China; 3 Department of Orthopedics, the People’s Hospital of Jiyuan, Jiyuan, 459000 Henan Province China; 4 Department of Medical Oncology, The Affiliated Cancer Hospital of Zhengzhou University and Henan Cancer Hospital, Zhengzhou, 450008 Henan Province China

**Keywords:** Tyrosine kinase inhibitor, Osteosarcoma, Soft tissue sarcoma, Adverse events, Pneumothorax

## Abstract

*Background* Previous studies have demonstrated the efficacy of apatinib and anlotinib for the treatment of sarcomas. However, more clinical data and evidence are needed to support clinical treatment selection and study design. Here, we evaluated the effectiveness and safety of these two drugs for the treatment of sarcomas. *Methods* We retrospectively reviewed the data of 110 patients with advanced osteosarcoma (*n* = 32) or soft tissue sarcoma (STS, *n* = 78) who received oral apatinib or anlotinib therapy during May 2016–February 2019 at two centers. Patients were divided into the apatinib and anlotinib groups. *Results* Among osteosarcoma patients, the objective response rates (ORRs) for the apatinib and anlotinib groups were 15.79% (3/19) and 7.69% (1/13), respectively. The disease control rates (DCRs) were 63.16% (12/19) and 30.77% (4/13), and the median progression-free survival (m-PFS) was 4.67 ± 3.01 and 2.67 ± 1.60 months, respectively. Among STS patients, ORRs for the apatinib and anlotinib groups were 12.24% (6/49) and 13.79% (4/29), respectively. The DCRs were 59.18% (29/49) and 55.17% (16/29), and m-PFS was 7.82 ± 6.90 and 6.03 ± 4.50 months, respectively. Regarding adverse events (AEs), apatinib was associated with a higher incidence of hair hypopigmentation and pneumothorax, while anlotinib was associated with a higher incidence of pharyngalgia or hoarseness. *Conclusion* Both apatinib and anlotinib were effective for the treatment of sarcomas. However, the effectiveness of the two drugs and associated AEs varied based on the histological type of sarcoma. These differences may be due to their different sensitivities to targets such as RET, warranting further study.

## Introduction

Sarcomas are malignancies of mesenchymal origin, of which more than 70 histological subtypes have been identified [1–3]. Sarcomas are relatively rare, with an annual incidence of fewer than 5 cases per 100,000 people, and these malignancies account for 1–2% of all adult cancers [1, 3]. Despite the rarity of sarcomas, more than 20,000 new cases of sarcoma are diagnosed annually in China [4]. Sarcomas can be subdivided into bone tumors (mostly osteosarcoma) and soft tissue sarcomas (STSs) according to pathological and histological features [5].

Surgical resection is the most important treatment option for sarcomas. However, 30–50% of sarcomas eventually recur or metastasize after surgery, and some patients present with metastases at the initial diagnosis [6, 7]. For metastatic or locally unresectable cases, doxorubicin-based chemotherapy is the first-line treatment. However, the response rates of advanced sarcomas to chemotherapy are only 14–48% [8, 9]. Therefore, more effective clinical treatments for advanced sarcomas are needed.

The emergence of small-molecule, multi-target tyrosine kinase inhibitors (TKIs) has advanced the treatment of sarcoma. Since the initial approval of pazopanib for STSs by the United States Food and Drug Administration (FDA) in 2012 [10], an increasing number of studies have demonstrated the efficacy of TKIs for the treatment of sarcomas [11, 12]. Apatinib is a small-molecule drug that potently and highly selectively inhibits the tyrosine kinase activity of vascular endothelial growth factor receptor 2 (VEGFR2) in vitro, and also inhibits the activities of VEGFR1, Kit, c-SRC, and RET tyrosine kinases [13]. This drug was approved by the Chinese FDA (CFDA) for the treatment of advanced gastric cancer in 2014 [14]. Anlotinib is a newly developed oral small-molecule TKI that targets VEGFR2, VEGFR3, Kit, VEGFR1, platelet-derived growth factor receptor (PDGFR)-α, and multiple fibroblast growth factor receptors (FGFR1, FGFR2, and FGFR3) [15]. This drug was approved by the CFDA for the treatment of advanced non-small cell lung cancer in 2018 [16].

Studies have revealed that apatinib and anlotinib display promising activity against sarcomas [17–19]. As the Affiliated People’s Hospital of Zhengzhou University and Affiliated Cancer Hospital of Zhengzhou University are two major sarcoma treatment centers in central China, we have treated many advanced sarcoma patients with multi-target TKIs. For several reasons, some patients were treated with apatinib, while others were treated with anlotinib. In this study, we retrospectively investigated these patients and studied the similarities and differences between patients treated with apatinib and anlotinib, with the aim of providing more evidence to support clinical treatment selection and clinical study design.

## Methods

### Patients and eligibility criteria

This was a retrospective study of patients treated at two hospitals: Affiliated People’s Hospital of Zhengzhou University and Affiliated Cancer Hospital of Zhengzhou University. The study was performed according to the principles and guidelines of the Declaration of Helsinki and approved by the Institutional Review Board of the Affiliated Cancer Hospital of Zhengzhou University. Patient enrollment began in May 2016 and ended in February 2019. The patient eligibility criteria included the following: 1) histologically proven osteosarcoma or STS; 2) age between 15 and 70 years; 3) confirmed ineligibility for radiotherapy or surgical treatment; 4) Eastern Cooperative Oncology Group Performance Status (ECOG PS) 0 or 1; 5) no history of treatment with other targeted drugs; 6) measurable lesions according to the Response Evaluation Criteria in Solid Tumors (RECIST) 1.1; and 7) acceptable hematologic (absolute neutrophil count ≥1.5 × 10^9^ cells/L, platelets ≥100 × 10^9^/L, and hemoglobin concentration ≥ 9 g/dL); hepatic (aspartate aminotransferase and alanine aminotransferase ≤2.5 × upper limit of normal [ULN], bilirubin ≤1.5 × ULN, and alkaline phosphatase ≤2.5 × ULN); and renal function (serum creatinine ≤1.5 × ULN, glomerular filtration rate ≥ 30 mL/min per 1.73 m^2^ according to the modified diet in renal disease abbreviated formula, and normal spot urine analysis results). This analysis was considered descriptive, and follow-up was extended until November 30, 2019.

### Treatment

According to the received treatment, patients were divided into the apatinib and anlotinib groups. In the apatinib group, patients received a once-daily oral dose of 500 mg apatinib. This apatinib dose was reduced to 250 mg per day for patients with intolerable adverse events (AEs). In the anlotinib group, patients received a once-daily oral dose of 12 mg anlotinib on days 1–14 of a 21-day cycle. This anlotinib dose was reduced to 10 mg per day for patients with intolerable AEs. Both drugs were administered continuously until intolerable AEs or progressive disease (PD) occurred. AEs were assessed using the National Cancer Institute Common Terminology Criteria for Adverse Events (NCI-CTCAE), version 4.0. If a severe AE occurred, apatinib or anlotinib administration was delayed for a maximum of 14 days to enable recovery.

### Evaluation

Tumor responses were evaluated every 2 months with computed tomography or magnetic resonance imaging. If a clear signal of PD was observed, evaluation was performed immediately. Tumor responses were evaluated according to the RECIST version 1.1 and were categorized as a complete response (CR), partial response (PR), stable disease (SD), or PD. The objective response rate (ORR) was defined as the sum of the rates of CR and PR. The disease control rate (DCR) was defined as the sum of the ORR and SD. Differences in the ORR, DCR, median progression-free survival (m-PFS), and AE incidence between the anlotinib and apatinib groups were also assessed. PFS was calculated from the date of the first dose of apatinib or anlotinib until the date of documented progression or death from any cause.

### Statistical analysis

Quantitative variables are presented as medians (ranges) or numbers of patients (percentages). PFS was estimated using the Kaplan–Meier method with a 95% confidence interval (CI). The survival curves were generated using GraphPad Prism 5.0 (La Jolla, CA, USA). Statistical analyses were performed using SPSS 21.0 software for Windows (IBM Corp., Armonk, NY, USA).

## Results

### Patient characteristics

A total of 121 patients received apatinib or anlotinib treatment during the study period. Eight patients were lost to follow-up; three patients dropped out for other reasons. Finally, 110 patients were enrolled, including 32 with osteosarcoma and 78 with STS.

The characteristics of the osteosarcoma patients are shown in Table 1. Nineteen and 13 patients received apatinib and anlotinib, respectively, and these groups had average ages of 22.42 ± 13.26 and 20.46 ± 11.15 years, respectively. Among patients in the apatinib group, 57.89% and 42.11% had an ECOG PS of 0 or 1, respectively. Among patients in the anlotinib group, 53.85% and 46.15% had an ECOG PS of 0 or 1, respectively. The primary lesions were distributed all over the body, although the most common sites were the femur, tibia, humerus, and axial skeleton. Most patients underwent primary lesion excision surgery (84.21% [16/19] and 84.62% [11/13] patients in the apatinib and anlotinib groups, respectively). The lung was the most frequent location of metastases (94.74% [18/19] and 92.31% [12/13] patients in the apatinib and anlotinib groups, respectively). The average elapsed time from the end of chemotherapy to the start of TKI treatment was 4.32 ± 2.81 and 4.62 ± 2.40 months in the apatinib and anlotinib groups, respectively.

**Table 1:** Basic characteristics of the two osteosarcoma groups

Characteristics	Apatinib group (*n* = 19)	Anlotinib group (*n* = 13)
Gender		
Male	10 (52.63%)	5 (38.46%)
Female	9 (47.37%)	8 (61.54%)
Age	22.42 ± 13.26	20.46 ± 11.15
ECOG PS		
0	11 (57.89%)	7 (53.85%)
1	8 (42.11%)	6 (46.15%)
Primary site		
Femur	6 (31.58%)	5 (38.46%)
Axial skeleton	2 (10.53%)	1 (7.69%)
Tibia	5 (26.32%)	4 (30.77%)
Humerus	3 (15.79%)	2 (15.38%)
Fibula	1 (5.26%)	0 (0.00%)
Other	1 (5.26%)	1 (7.69%)
Radial	1 (5.26%)	0 (0.00%)
Excision of primary lesion		
No	3 (15.79%)	2 (15.38%)
Yes	16 (84.21%)	11 (84.62%)
Metastatic site		
Only lung	14 (73.68%)	10 (76.92%)
Only bone	1 (5.26%)	1 (7.69%)
Both bone and lung	4 (21.05%)	2 (15.38%)
Previous MAP/I chemotherapy		
No	1 (5.26%)	0 (0.00%)
Yes	18 (94.74%)	13 (100.00%)
Previous other chemotherapy		
No	17 (89.47%)	12 (92.31%)
Yes	2 (10.53%)	1 (7.69%)
Time interval (months)	4.32 ± 2.81	4.62 ± 2.40

**Notes:** Data are presented as numbers (percentages) or means ± standard deviations

**Abbreviations**: ECOG PS, Eastern Cooperative Oncology Group performance status; MAP/I, high-dose methotrexate, doxorubicin, cisplatin, and/or ifosfamide; Time interval, time interval between the end of chemotherapy and oral apatinib or anlotinib administration

The basic characteristics of the STS patients are listed in Table 2. Forty-nine and 29 patients received apatinib and anlotinib, respectively. The average ages were 41.10 ± 14.20 and 41.86 ± 14.27 years in the apatinib and anlotinib groups, respectively. All patients had a good performance status (ECOG PS 0/1). The histological subtypes included undifferentiated pleomorphic sarcoma (UPS, *n* = 15); synovial sarcoma (*n* = 14); leiomyosarcoma (*n* = 9); liposarcoma (*n* = 7); malignant peripheral nerve sheath tumor (*n* = 5); angiosarcoma (n = 5); epithelioid sarcoma (n = 5); rhabdomyosarcoma (*n* = 4); fibrosarcoma (n = 5); alveolar soft part sarcoma (*n* = 3); clear cell sarcoma (n = 3); malignant granulosa cell tumor (*n* = 1); and primitive neuroectodermal tumor (n = 1). Most patients underwent primary lesion excision surgery (83.67% [41/49] patients in the apatinib group and 86.21% [25/29] patients in the anlotinib group). The lung was the most frequent site of metastasis (87.76% [43/49] patients in the apatinib group and 86.21% [25/29] patients in the anlotinib group), and all patients had received at least one cycle of chemotherapy previously. The average elapsed time from the end of chemotherapy to the start of TKI treatment was 4.78 ± 2.04 and 4.55 ± 2.21 months in the apatinib and anlotinib groups, respectively.

**Table 2:** Basic characteristics of the two soft tissue sarcoma groups

Characteristics	Apatinib group (*n* = 49)	Anlotinib group (*n* = 29)
Gender		
Male	27 (55.10%)	15 (51.72%)
Female	22 (44.90%)	14 (48.28%)
Age	41.10 ± 14.20	41.86 ± 14.27
ECOG PS		
0	24 (48.98%)	15 (51.72%)
1	25 (51.02%)	14 (48.28%)
Histological type		
UPS	10 (20.41%)	5 (17.24%)
Synovial sarcoma	7 (14.29%)	7 (24.14%)
Leiomyosarcoma	6 (12.24%)	3 (10.34%)
Liposarcoma	5 (10.20%)	2 (6.90%)
MPNST	4 (8.16%)	1 (3.45%)
Angiosarcoma	4 (8.16%)	1 (3.45%)
Clear cell sarcoma	3 (6.12%)	0 (0.00%)
Epithelioid sarcoma	3 (6.12%)	2 (6.90%)
Rhabdomyosarcoma	3 (6.12%)	1 (3.45%)
Fibrosarcoma	2 (4.08%)	3 (10.34%)
ASPS	1 (2.04%)	3 (10.34%)
Malignant granulosa cell tumor	1 (2.04%)	0 (0.00%)
PNET	0 (0.00%)	1 (3.45%)
Locally unresectable or metastatic		
Locally unresectable	7 (14.29%)	5 (17.24%)
Metastatic	42 (85.71%)	24 (82.76%)
Primary site		
Extremities	34 (69.39%)	20 (68.97%)
Trunk	15 (30.61%)	9 (31.03%)
Excision of primary lesion		
No	8 (16.33%)	4 (13.79%)
Yes	41 (83.67%)	25 (86.21%)
Metastatic site		
Lungs	43 (87.76%)	25 (86.21%)
Other	6 (12.24%)	4 (13.79%)
Lines of previous chemotherapy		
1	33 (67.35%)	17 (58.62%)
2	12 (24.49%)	11 (37.93%)
3	4 (8.16%)	1 (3.45%)
Time interval (months)	4.78 ± 2.04	4.55 ± 2.21

**Notes:** Data are presented as numbers (percentages) or means ± standard deviations

**Abbreviations**: ECOG PS, Eastern Cooperative Oncology Group performance status; UPS, undifferentiated pleomorphic sarcoma; MPNST, malignant peripheral nerve sheath tumor; ASPS, alveolar soft part sarcoma; PNET, primitive neurotodermal tumor; Time interval, time interval between the end of chemotherapy and oral apatinib or anlotinib administration

## Clinical effectiveness

### Osteosarcoma patients

**Fig. 1:**
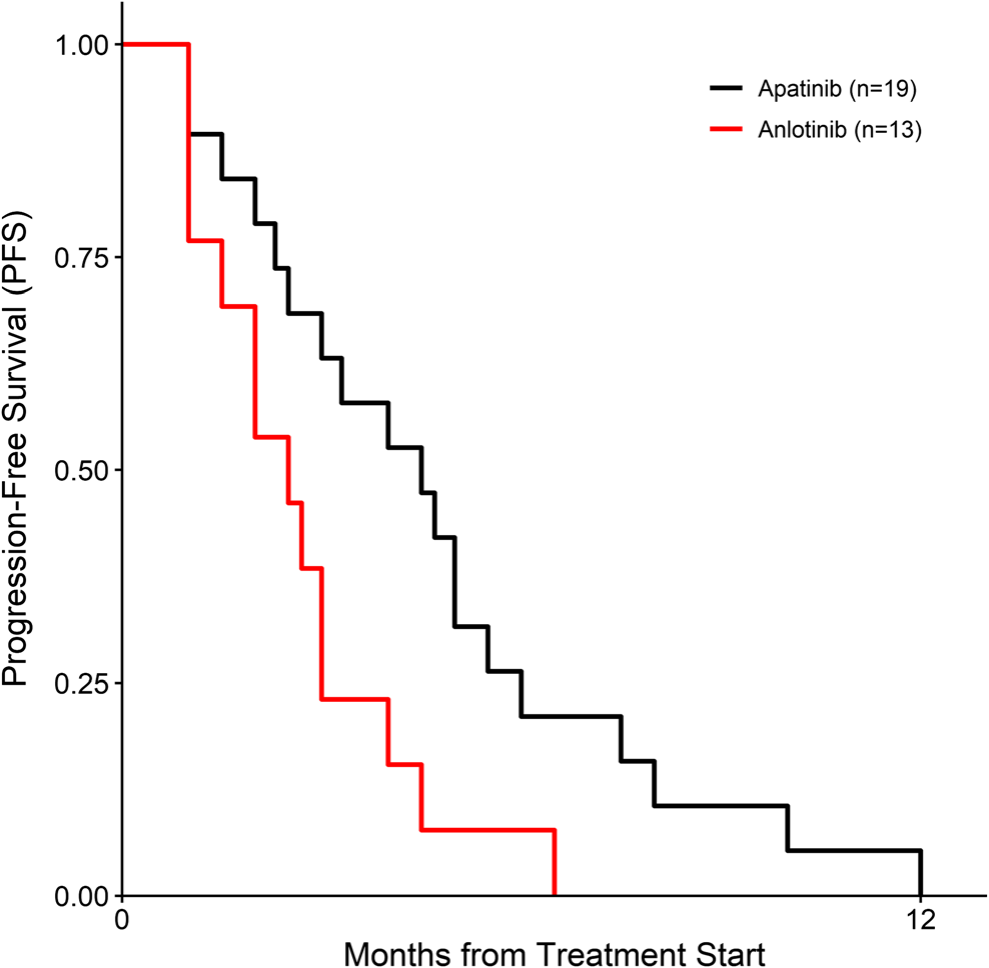
Kaplan–Meier estimates of progression-free survival among patients with osteosarcoma after treatment with apatinib or anlotinib

None of the 32 osteosarcoma patients achieved CR. The apatinib group had an ORR of 15.79%, DCR of 63.16%, and m-PFS of 4.67 ± 3.01 months. The anlotinib group had an ORR of 0.00%, DCR of 23.08%, and m-PFS of 2.67 ± 1.60 months (Table 3, Fig. 1).

**Table 3:** Clinical efficacy of apatinib and anlotinib in osteosarcoma

Characteristics	Apatinib group (n = 19)	Anlotinib group (n = 13)
ORR (%)	3 (15.79%)	1 (7.69%)
DCR (%)	12 (63.16%)	4 (30.77%)
m-PFS (months)	4.67 ± 3.01	2.67 ± 1.60

**Notes:** Data are presented as numbers (percentages) or means ± standard deviations

**Abbreviations:** ORR, objective response rate; DCR, disease control rate; m-PFS, median progression-free survival

### STS patients

**Fig. 2:**
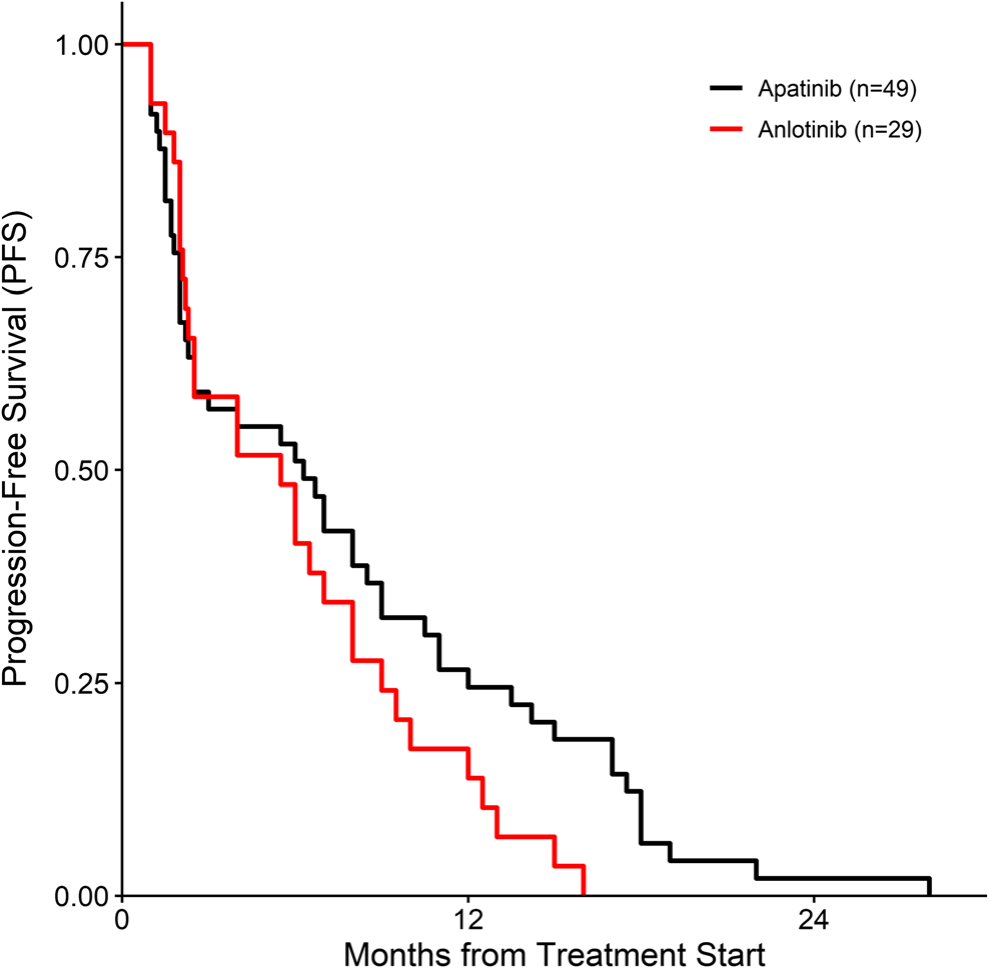
Kaplan–Meier estimates of progression-free survival among patients with soft tissue sarcoma after treatment with apatinib or anlotinib

One UPS patient in the apatinib group achieved a CR (Table 4). The apatinib group had an ORR of 12.24%, DCR of 59.18%, and m-PFS of 7.82 ± 6.90 months. The anlotinib group had an ORR of 13.79%, DCR of 55.17%, and m-PFS of 6.03 ± 4.50 months (Table 5, Fig. 2).

**Table 4:** Responses of various histological subtypes to treatment

Histological subtype	Apatinib group (*n* = 49)				Anlotinib group (*n* = 29)			
	CR	PR	SD	PD	CR	PR	SD	PD
UPS	1	3	4	2		1	1	3
Synovial sarcoma		1	4	2		1	4	2
Leiomyosarcoma		1	2	3		1	2	
Liposarcoma			3	2			1	1
MPNST			1	3				1
Angiosarcoma		1	2	1				1
Clear cell sarcoma			1	2				
Epithelioid sarcoma			1	2			1	1
Rhabdomyosarcoma			2	1				1
Fibrosarcoma			1	1			1	2
ASPS		1				1	2	
MGCT				1				
PNET								1
Total	1	7	21	20		4	12	13

**Abbreviations:** CR, complete response; PR, partial response; SD, stable disease; PD, progressive disease; UPS, undifferentiated pleomorphic sarcoma; MPNST, malignant peripheral nerve sheath tumor; ASPS, alveolar soft part sarcoma; MGCT, malignant granulosa cell tumor; PNET, primitive neurotodermal tumor

**Table 5:** Clinical efficacy of apatinib and anlotinib in soft tissue sarcoma

Characteristics	Apatinib group (n = 49)	Anlotinib group (n = 29)
ORR (%)	6 (12.24%)	4 (13.79%)
DCR (%)	29 (59.18%)	16 (55.17%)
m-PFS (months)	7.82 ± 6.90	6.03 ± 4.50

**Notes:** Data are presented as numbers (percentages) or means ± standard deviations

**Abbreviations:** ORR, objective response rate; DCR, disease control rate; m-PFS, median progression-free survival

### Toxicity evaluation

AEs appeared to be more prevalent in the apatinib group than in the anlotinib group (Table 6). Most AEs were grade 1 or 2, although a few were grade 3 or 4, and no drug-related deaths occurred. Some AEs occurred more frequently in the apatinib group, including hair hypopigmentation and pneumothorax. Pharyngalgia or hoarseness was more frequent in the anlotinib group (Table 6).

**Table 6:** Adverse events

Events	Apatinib group				Anlotinib group			
	Osteosarcoma (19 cases)		STS (49cases)		Osteosarcoma (13 cases)		STS (29 cases)	
	any grade	grade > 2	any grade	grade > 2	any grade	grade > 2	any grade	grade > 2
Rash or hand-foot syndrome	12 (63.16%)	2 (10.53%)	31 (63.27%)	4 (8.14%)	6 (46.15%)	1 (7.70%)	16 (55.17%)	1 (3.45%)
Hair hypopigmentation	9 (47.37%)	0 (0%)	21 (42.86%)	0 (0%)	0 (0%)	0 (0%)	0 (0%)	0 (0%)
Hypertension	8 (42.11%)	1 (5.26%)	25 (51.02%)	7 (14.29%)	6 (46.15%)	1 (7.70%)	16 (55.17%)	2 (6.70%)
Anorexia	8 (42.11%)	1 (5.26%)	17 (34.69%)	2 (4.08%)	3 (23.77%)	0 (0%)	9 (31.03%)	0 (0%)
Diarrhea or abdominal pain	7 (36.84%)	1 (5.26%)	16 (32.65%)	2 (4.08%)	5 (38.46%)	1 (7.70%)	14 (48.28%)	1 (3.45%)
Pneumothorax	6 (31.58%)	3 (15.79%)	3 (6.12%)	1 (2.04%)	2 (15.38%)	0 (0%)	2 (6.70%)	1 (3.45%)
Weight loss	6 (31.58%)	1 (5.26%)	14 (28.57%)	0 (0%)	4 (30.77%)	0 (0%)	8 (29.59%)	0 (0%)
Fatigue	6 (31.58%)	0 (0%)	15 (30.61%)	0 (0%)	5 (38.46%)	0 (0%)	9 (31.03%)	0 (0%)
Wound-healing problems	2 (10.53%)	0 (0%)	4 (8.14%)	0 (0%)	1 (7.70%)	0 (0%)	1 (3.45%)	0 (0%)
Arthralgia	2 (10.53%)	0 (0%)	5 (10.20%)	0 (0%)	1 (7.70%)	0 (0%)	1 (3.45%)	0 (0%)
Hemoptysis	2 (10.53%)	0 (0%)	1 (2.04%)	0 (0%)	0 (0%)	0 (0%)	1 (3.45%)	0 (0%)
Oral mucositis	2 (10.53%)	0 (0%)	5 (10.20%)	1 (2.04%)	2 (15.38%)	0 (0%)	4 (13.79%)	0 (0%)
Pharyngalgia or hoarseness	0 (0%)	0 (0%)	0 (0%)	0 (0%)	7 (53.85%)	0 (0%)	15 (51.72%)	0 (0%)

**Notes:** Data are presented as numbers (percentages)

**Abbreviations:** STS, soft tissue sarcoma

## Discussion

The treatment of advanced sarcomas and efficacies of therapies have long remained stagnant. The current four-drug combination chemotherapy regimen was established as the first-line treatment for advanced osteosarcoma in the 2000s [7] and is associated with m-PFS of <4 months [12]. For advanced STS, doxorubicin-based chemotherapy has been administered as the first-line treatment for decades [6], and is associated with m-PFS of 5–8 months [9]. However, this scenario has changed with the advent of TKIs. TKIs target tyrosine kinases, which are key mediators of intracellular signaling cascades. Consequently, aberrations in these proteins have been implicated as drivers of oncogenesis via the dysregulation of fundamental cellular processes, including proliferation, migration, and apoptosis [20]. TKI-based therapy has led to significant advances in the treatment of many malignancies. All TKIs with promising preclinical and clinical effectiveness against sarcoma, including apatinib, anlotinib, axitinib, imatinib, pazopanib, regorafenib, sorafenib, and sunitinib, target multiple angiogenic and growth-promoting receptor tyrosine kinases, as shown in Table 7.

**Table 7:** Targets and clinical outcomes of multi-target TKIs that are effective for sarcoma treatment

TKIs	Targets in order of selectivity	Clinical outcomes of therapy for sarcoma^a^
Anlotinib	VEGFR2 < VEGFR3 < KIT < VEGFR1 < < PDGFRβ [21]	166 patients with refractory metastatic STS [19] • ORR 13% • m-PFS 5.6 months (m) • m-OS 12 m
Apatinib	VEGFR2 < RET < VEGFR1 < KIT < C-SRC [13]	64 patients with stage IV bone and soft tissue sarcoma [22] • ORR 17% • m-PFS 7.9 m • m-OS 17 m
Axitinib	PDGFRα < PDGFRβ < KIT < VEGFR1 < VEGFR2 < < FGFR2 < RET < VEGFR3 < FGFR3 < FGFR1 < < MET << NTRK1 [20]	64 patients with progressive advanced solitary fibrous tumor [23] • ORR 41% • m-PFS 5.1 m • m-OS 25 m
Pazopanib	VEGFR1 < VEGFR2 < VEGFR3 < PDGFRα < KIT < PDGFRβ < FGFR3 < FGFR1 [24]	246 patients with metastatic STS [25] • ORR 9% • m-PFS 4.6 m • m-OS 12.5 m
Regorafenib	RET < PDGFRβ < PDGFRα < VEGFR1 < ABL1 < KIT < VEGFR3 < VEGFR2 < < NTRK3 [26]	182 patients with advanced STS [27] • Regorafenib has an important clinical antitumor effect in non-adipocytic STSs, improving PFS.
Sorafenib	RET < PDGFRβ < VEGFR2 < VEGFR3 < PDGFRα < KIT < ABL1 < < NTRK3 < < NTRK2 < < FGFR2 < FGFR1 < < FGFR3 < < FGFR4 < NTRK1 [28]	145 patients with metastatic or recurrent sarcomas [29] • As a single agent, sorafenib has activity against angiosarcoma and minimal activity against other sarcomas.
Sunitinib	PDGFRB < KIT < PDGFRA < VEGFR2 < VEGFR1 < RET << VEGFR3 < < NTRK1 < < ALK << ABL1 < FGFR3 < < FGFR1/2 < NTRK2 < < FGFR4 = SRC < < NTRK3 < < MET [20]	48 patients with relapsed or refractory sarcomas [30] • A 3-month PFS rate of >40% suggests that sunitinib malate at least has activity against liposarcomas and leiomyosarcomas.

^a^Only the studies with the largest sample size or the highest credibility are listed

**Abbreviations:** STS, soft tissue sarcoma; ORR, objective response rate; m-PFS, median progression-free survival; m-OS, median overall survival

Apatinib and anlotinib, the only two domestically developed multi-target TKIs, have been marketed and used widely for the treatment of advanced sarcomas in China [17, 18, 21]. Although many clinical studies of apatinib and anlotinib have begun to recruit patients with sarcoma (http://www.chictr.org.cn), this study is the first to simultaneously investigate the effectiveness and safety of these two drugs in patients with advanced sarcomas. In this retrospective observational study, we found that both apatinib and anlotinib were effective for the treatment of sarcomas. However, the effectiveness of the two drugs and corresponding AEs varied based on the histological type of sarcoma. Apatinib appeared to be more effective in osteosarcoma, and it was associated with higher incidences of hair hypopigmentation and pneumothorax. On the other hand, anlotinib was associated with a higher incidence of pharyngalgia or hoarseness.

Our finding that apatinib was effective for the treatment of osteosarcoma was consistent with the results of previous studies. To date, at least five studies have demonstrated the effectiveness of apatinib for treatment of osteosarcoma [17, 22–25]. While none have reported the effectiveness of anlotinib. Although both drugs are multi-target TKIs, they differ with regard to the therapeutic effectiveness against osteosarcoma. We screened clinical trials registered with ClinicalTrials.gov for nearly all small-molecule TKIs and found that only three TKIs (apatinib, regorafenib, and sorafenib) have been identified as promising for the treatment of osteosarcoma [12, 22, 26]. These three TKIs share a distinctive sensitivity for VEGFR2 and RET (Tables 7 and 8) [13, 27–30], suggesting that RET, like VEGFR2, may be an important specific target in the treatment of osteosarcoma.

**Table 8:** Sensitive targets of anlotinib, apatinib, sorafenib, and regorafenib

Kinases	IC_50_ (nM, mean)			
	Anlotinib	Apatinib	Sorafenib	Regorafenib
VEGFR1	26.9	70	–	13
VEGFR2	0.2	1	4	4.2
VEGFR3	0.7	–	20	46
KIT	14.8	429	68	7
PDGFRα	167	>1000	57	22
RET	n.d.	13	0.4	1.5
C-SRC	–	530	–	–
FGFR1	40.4	–	580	202

**Abbreviations:** IC_50_, half maximal inhibitory concentration; nM, nmol/l; n.d., not determined

In contrast, we did not observe a difference in the therapeutic effects of apatinib and anlotinib in patients with STS. Several other studies have also demonstrated the effectiveness of both TKIs for the treatment of STS [19, 21, 25, 31, 32]. However, these two TKIs differ with respect to therapeutic effectiveness for specific subtypes of STS. For example, we demonstrated different effectiveness of these drugs for the treatment of UPS and leiomyosarcoma (Table 4). Nonetheless, the large number of STS subtypes falsely suggests that these two TKIs have similar efficacies. We speculate that these different therapeutic effects of apatinib and anlotinib on different subtypes of STS involve a fundamental difference in target sensitivity (Table 8). Further research is required to elucidate the mechanisms of action beyond the currently identified targets.

**Fig. 3:**
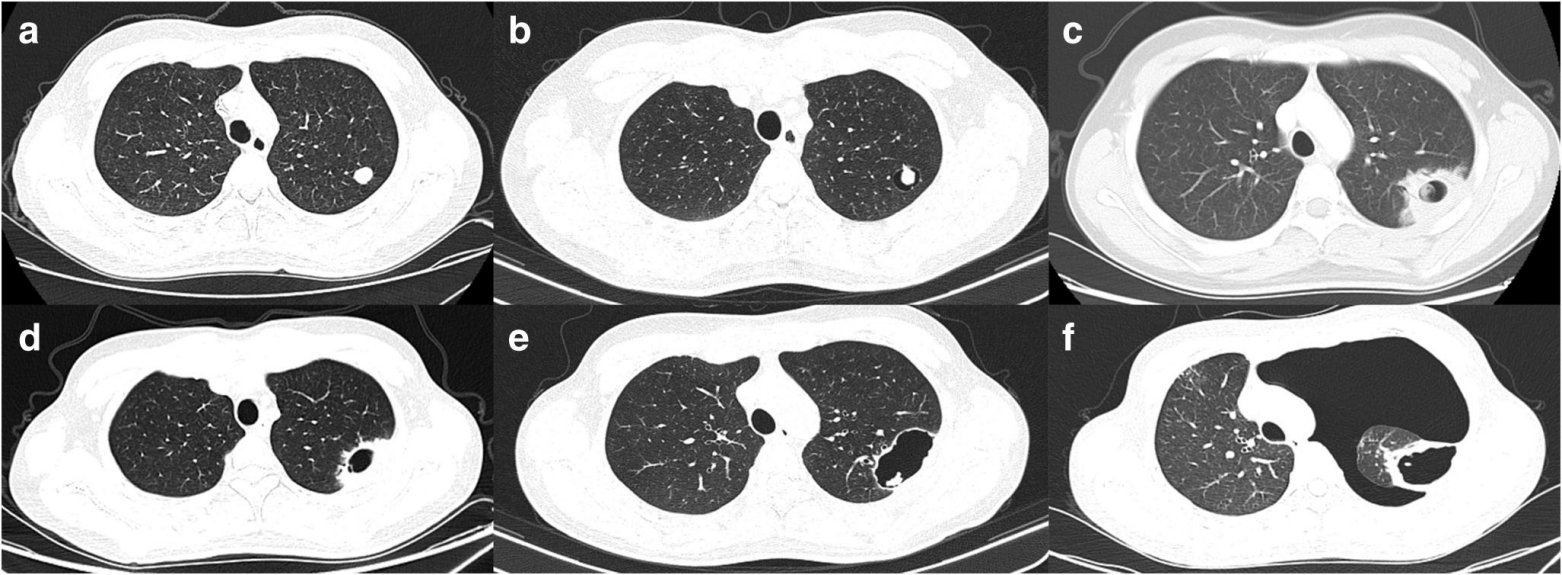
Typical development of pneumothorax after apatinib treatment in an osteosarcoma patient. Computed tomography scans were obtained **a** at treatment initiation, **b** 1 month, **c** 2 months, **d** 3 months, **e** 5 months, and **f** 6 months after treatment

We further observed an increased incidence of pneumothorax in patients with osteosarcoma who were treated with apatinib rather than with anlotinib. In such cases, the basic pathological process of pneumothorax involved necrosis, cavitation in the metastatic lung lesions, and finally pneumothorax formation (as shown in Fig. 3), consistent with other reports of osteosarcoma [31, 32]. However, apatinib was not reported to induce this AE in patients with other types of malignancies (i.e., non-sarcoma) [14, 33]. Moreover, significant increases in pneumothorax were not observed in response to sorafenib and regorafenib, which are as effective as apatinib for osteosarcoma [12, 26]. We also observed that the incidences of hair hypopigmentation and pharyngalgia or hoarseness differed between the apatinib and anlotinib groups. We speculate that these differences could be attributable to the targets of these TKIs (Table 8).

The main limitations of this study include the retrospective design, the absence of a control group, and the abundance of uncommon sarcoma subtypes. A registered clinical study on the efficacy of apatinib versus anlotinib for different subtypes of sarcoma must be conducted to obtain more accurate and reliable evidence. Moreover, the mechanism by which apatinib induces pneumothorax during the treatment of pulmonary metastatic osteosarcoma requires further study. More importantly, studies on the role and mechanism of RET in the treatment of osteosarcoma by multi-target TKIs may yield unexpected results.

In conclusion, apatinib and anlotinib were both effective for the treatment of sarcomas. The effectiveness of the two drugs and associated AEs varied based on the histological type of sarcoma. These differences may be due to their different sensitivities to targets such as RET, warranting further study.
